# Evaluation Model of Innovation and Entrepreneurship Ability of Colleges and Universities Based on Improved BP Neural Network

**DOI:** 10.1155/2022/8272445

**Published:** 2022-08-02

**Authors:** Shixiao Li

**Affiliations:** Employment Guidance Teaching and Research Department, Henan Institute of Technology, Xinxiang 453000, Henan, China

## Abstract

Entrepreneurship education activities in colleges and universities play an important role in improving students' innovation ability. Therefore, this paper has important practical value to evaluate the innovation and entrepreneurship ability of college students. At present, most studies use qualitative research methods, which is inefficient. Even if quantitative analysis is adopted, it is mostly linear analysis, which is inconsistent with the actual situation. In order to improve the application level of genetic algorithm to the innovation and entrepreneurship ability of universities based on BP neural network, this paper studies the evaluation model of innovation and entrepreneurship ability of universities. Based on the simple analysis of the current situation of university innovation and entrepreneurship ability evaluation and the application progress of BP neural network, combined with the actual situation of university innovation and entrepreneurship, this paper constructs the innovation and entrepreneurship evaluation index, uses BP neural network to build the evaluation model, and uses genetic algorithm to optimize and improve the shortcomings of BP neural network. Then, the experimental analysis and application design are carried out. The results show that the improved algorithm is basically consistent with the predicted value, small error, and fast convergence. When it is used in the evaluation of innovation and entrepreneurship ability, quantitative analysis results can be obtained, which provides a certain reference for the development of enterprises.

## 1. Introduction

Colleges and universities should implement and improve the preferential policies for innovation and entrepreneurship. Colleges and universities across the country should thoroughly implement the “entrepreneurship guidance plan for college students.” They should actively work with relevant departments to further strengthen the implementation of policies and implement various support policies, such as entrepreneurship guarantee loans, tax cuts, and fees reductions for small and micro enterprises and entrepreneurship training subsidies. The level of curriculum reform and construction is not only an important symbol to measure the level and quality of running a university, but also an important resource to create a university brand. The important principles of curriculum system reform should fully reflect the requirements of talent training objectives. Different types of colleges and universities have different curriculum systems due to different training objectives. Curriculum system is an important means for colleges and universities to realize their own educational ideas and cultivate applied and compound talents. This paper analyzes the challenges faced by China's higher education curriculum system and draws on the successful experience of foreign universities' curriculum and innovative talent training. This paper puts forward some suggestions on the innovation of the professional curriculum system based on the demand of the talent market in China's higher education.

In recent years, the state has been fully advocating innovative ideas. All industries in China, including the education industry, have rapidly formed a new trend of innovative social development, and innovation has formed a public consensus. In order to improve the public's innovation awareness and ability, higher education school across the country have begun to build education courses in order to adapt to social development [1]. However, from the existing results, students' innovation and entrepreneurship activities have not been greatly improved. There are still some problems in teaching, such as backward teaching ideas, lack of innovation platform, and insufficient guidance ability [2]. Although there are many reasons for its formation, the lack of evaluation system is one of the important influencing factors [3]. Xie has evaluated the innovation and entrepreneurship in higher education school, recognized its importance and necessity, and also pointed out the shortcomings and improvement strategies in education, but most of them use the method of questionnaire survey, which lacks objectivity. In quantitative analysis, BP neural network is widely used. Neural network is one of the algorithms to simulate brain thinking and find the rules from big data [4]. Doblinger et al. put forward and evaluated the value creation mechanism of network resources of different types of start-up partners and highlighted the unique resources provided by government partners for clean technology start-ups [5]. Among all kinds of evolutionary algorithms, genetic algorithm is a special one. It can be combined with other algorithms to simulate the natural survival of the fittest. The characteristics of global optimization make up for the shortcomings of many algorithms.

This paper is based on the simple analysis of the current situation of innovation and entrepreneurship ability evaluation in colleges and universities and the application progress of BP neural network. Combined with the actual situation of innovation and entrepreneurship in colleges and universities, this paper constructs the evaluation index of innovation and entrepreneurship. BP neural network is used to build the evaluation model, and genetic algorithm is used to optimize and improve the shortcomings of BP neural network. Then, the experimental analysis and application design are carried out. The innovation contribution lies in the construction of the evaluation index system. BP neural network model is used to improve the operability of the evaluation. In view of the shortcomings of BP neural network, genetic algorithm is used to optimize the evaluation model. The algorithm continues random optimization, so there will be no local minimum. The improved algorithm retains the advantages of BP learning algorithm and can avoid falling into extreme value. The improved algorithm is basically consistent with the predicted value, small error, and fast convergence. When it is used in the evaluation of innovation and entrepreneurship ability, quantitative analysis results can be obtained, which provides a certain reference for the development of enterprises.

This paper analyzes the design and application of efficient innovation and entrepreneurship capability model based on improved BP network. The study is divided into four parts: first, BP learning algorithm and the chapter arrangement of this research; the second chapter introduces the research progress of teaching evaluation and the application of BP neural network and summarizes the shortcomings of the current research. The third chapter puts forward the index system and calculates the weight. Based on this, a BP neural network model is constructed to evaluate innovation and entrepreneurship ability. According to the shortcomings of BP neural network, genetic algorithm is used to improve it. Based on improving the characteristics of global convergence, the diversity of population is also used. The fourth chapter carries out simulation analysis and experimental analysis on the evaluation model of university innovation and entrepreneurship ability constructed in this paper and determines the convergence and error changes under different algorithms through simulation analysis. The output result of the improved algorithm using genetic algorithm is basically similar to the expected result, which can reduce the number of iterations, reduce the error change range, and run more stably. In the overall evaluation, the comprehensive ability evaluation result is 0.736, indicating that there is room for improvement, and some achievements have been made. The overall level is above the medium level.

## 2. State of the Art

With the severe employment situation and the improvement of national innovation consciousness, major higher education schools have carried out education and made some progress. Many scholars have evaluated and analyzed the ability. For example, Yujie et al. discussed the evaluation model in the research, thought that the fuzzy evaluation method was more reasonable, proposed to establish a multilevel simulation comprehensive evaluation, combined it with the graduation project to improve the hierarchy of teaching, and used the fuzzy algorithm to improve the curriculum system [6]. Jiang et al. also combined other algorithms with BP algorithm and applied it to the field of economics. For example, Jiang et al. combined particle swarm optimization algorithm with BP learning algorithm in their research, praised the improved model, and applied it to the comprehensive analysis of stocks and rising composite index. It was found that the improved algorithm has higher accuracy in prediction and reduced error [7]. The application of BP learning algorithm not only is reflected in evaluation, but also can be used in decision-making model. Yuan and Li selected the principal component analysis method when studying the index system established in the higher education quality model, took 12 indexes from 50 countries as the index evaluation system, extracted a total of 4 principal component factors that play a leading role, took these indexes as the decision-making standard, and then used BP learning algorithm to establish the evaluation and decision-making model, which has a good evaluation and prediction effect [8]. Chang et al. proposed a new method of undergraduate education quality evaluation based on algorithmic stress test and constructed an open index database composed of 4 dimensions and 19 variables such as teaching attitude, teaching content, teaching methods, and teachers' basic characteristics [9]. Han et al. used the theory of fuzzy mathematics to construct a fuzzy similarity matrix to describe the relationship between factors and judgment [10]. Wang et al. took the Internet of Things system as the research object in their research, measured the performance of different algorithms, respectively, compared and analyzed the BP learning algorithm with the MP algorithm based on BP NNA, measured the parameters of the transmission layer and application layer, and believed that the improved algorithm could optimize the parameters and shorten the running time [11].

To sum up, we can see that there are many researches. Most of them use qualitative research and analysis methods, use questionnaire to analyze the current situation of innovation and entrepreneurship, and put forward deficiencies and improvement strategies. This objective evaluation method is extremely arbitrary, there is no unified weight, the main influencing factors cannot be determined, and the pertinence is not strong. In addition, in the evaluation, the particularity of innovation and entrepreneurship education itself is not taken into account. The evaluation of innovation and entrepreneurship ability is not only the participation of schools and teachers, but also the full participation of students. Most of the existing studies are the same as the evaluation of other courses, ignoring the value of practice and not analyzing the evaluation of students. Although many scholars have used BP neural network algorithm to study teaching and improved BP neural network algorithm to some extent, it has strong objectivity and is rarely applied to evaluation.

## 3. Methodology

### 3.1. Design of Entrepreneurship Evaluation Index System

**Figure 1:**
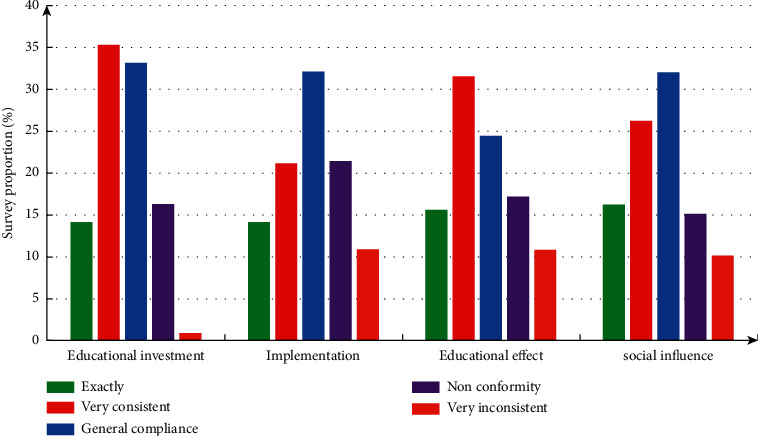
Relevant investigation.

The evaluation system covers educational investment, educational environment, educational effect, and social impact. The survey results are shown in Figure 1. These indicators are basically in line with the scope. Educational background mainly refers to the conditions for higher education school to carry out public guidance innovation and entrepreneurship theme activities. There are both internal environmental reasons and many external factors. The internal environment indicators include the setting of school functional departments, school running philosophy, innovative education incentive measures, the use of funds, the construction of education base, and teacher training. The off-campus environment covers two indicators: government support and local innovation and entrepreneurship activities.

**Figure 2:**
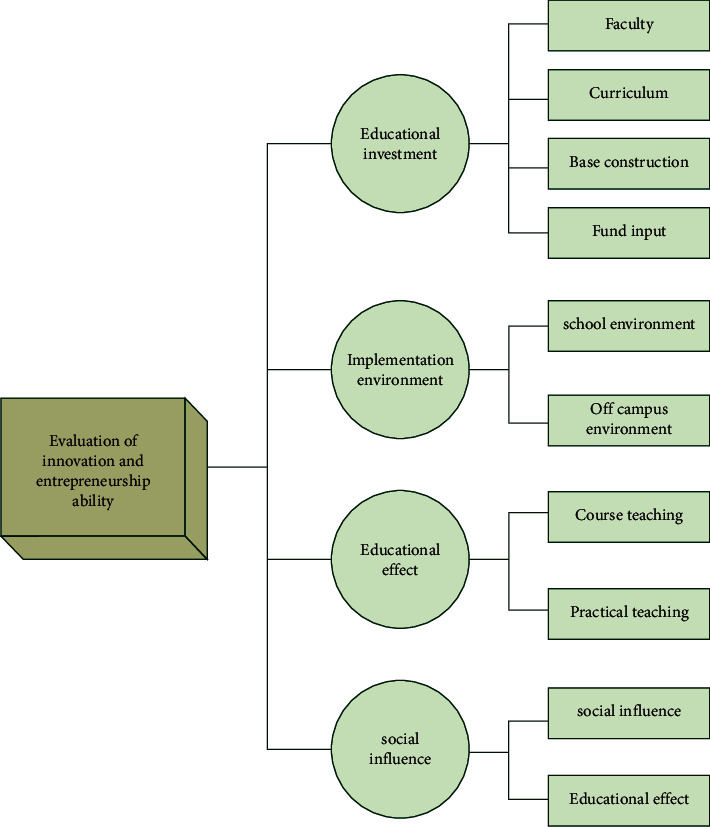
Evaluation index of ability of colleges.

The specific indicators are shown in Figure 2. There are 4 primary indicators, 10 secondary indicators, and 36 tertiary indicators. Education investment indicators can reflect the investment and allocation of resources. The resources include four indicators: teachers' strength indicators, base construction indicators, curriculum indicators, and fund investment. Among them, the input of teachers covers four indicators: the proportion of teacher resources input, the proportion of middle and senior professional titles of school staff, the proportion of teacher quality, and the proportion of teacher training skills [12]. The fund investment covers four indicators: teacher training fund, construction fund, and practice fund. The curriculum covers five indicators: basic curriculum arrangement, practical curriculum arrangement, professional curriculum arrangement, municipal excellent curriculum arrangement, and school-based resource development. The base platform construction covers three indicators: the number of bases, the number of entrepreneurship platforms, and the number of incubation bases. Education process is the core content of evaluation. The evaluation covers two parts: practice and curriculum teaching. Practice teaching covers four indicators: competition, training, number of activities, and student participation [13]. The course teaching evaluation covers five indicators: teaching methods, design, teaching contents, lectures, and the number of special seminars. Educational achievements reflect the feedback of innovation and entrepreneurship education results, mainly covering two secondary indicators, that is, social impact and educational effect. Social impact adopts three indicators: students' participation in small and micro enterprises, employment, and patents. The educational achievements reflect the school level, covering three indicators: the award-winning situation of innovation and entrepreneurship theme competitions held by colleges and universities, the situation of outstanding graduates and alumni, and social popularity and influence.

In the calculation of index weight, it is necessary to investigate through experts and compare the importance of indicators. The statistical average method is based on the relative importance coefficient given by the selected experts to each evaluation index. The arithmetic mean value is calculated, respectively, and the calculated mean value is used as the weight of each index. The basic steps are as follows: the first step is to determine experts. Generally, experts with practical work experience, solid theoretical foundation, fairness, justice, and noble morality in the industry or field are selected. The second step is preliminary evaluation by experts. Submit the indicators with undetermined weights to each expert, and ask the experts to independently give the weight values of each indicator without external interference. The third step is to recycle expert opinions. Take back the data of each expert, and calculate the weighted mean value and standard of each index. The fourth step is to calculate the average weight of each indicator. If there is a large difference in the judgment of the importance of indicators, further consultation shall be conducted by using the survey method to adjust the weight of indicators. The greater the weight value, the greater the importance. In the calculation of weight judgment matrix vector, the square root method is used to realize the normalization of each evaluation index data to calculate the weight vector. The formula is as follows:

(1) $$\begin{array}{c} W_{i}=\frac{{\bar{W}}_{i}}{\sum_{i=1}^{n}{\bar{W}}_{i}}. \end{array}$$

According to the same method, calculate the weight vector of the secondary index to the primary index and the weight vector of the tertiary index to the secondary index. Synthesize the weights of each composite weight of the target layer.

### 3.2. Evaluation Using the BP Neural Network Model

**Figure 3:**
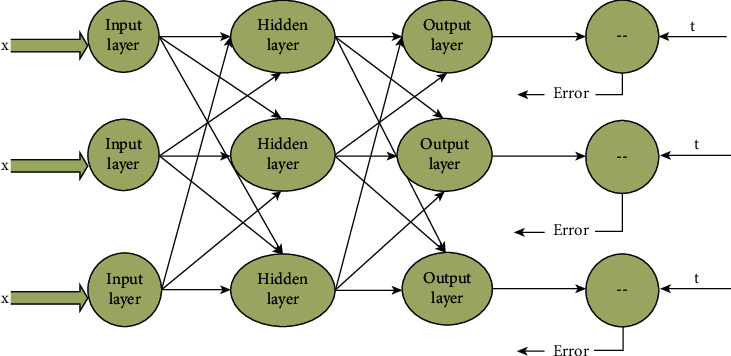
Structure diagram.

At present, most of the commonly used evaluation methods belong to qualitative analysis, which is inefficient. These methods can only get a general evaluation result, which does not greatly promote the whole teaching. The evaluation methods are not combined with the actual teaching situation. The existing teaching data mining is obviously insufficient, and the analysis results are not objective enough. In the evaluation of teaching activities and teaching effects in colleges and universities, the purpose of the various evaluation models established is ultimately to revolve around one purpose, that is, to improve the teaching level. Among all kinds of teaching methods, BP neural network algorithm is a kind of algorithm with high fault tolerance. Therefore, it is considered to apply this algorithm to the evaluation of teaching in higher education school [13]. The neural network model is shown in Figure 3. The input layer and output layer can be selected by themselves. Multivariable or univariate can be used in the calculation. Without considering the influence of other systems, it can deal with qualitative problems and quantitative calculation [14].

BP neural network structure belongs to multilayer feedforward network. In solving the problem, this model needs to collect various characteristic data of the evaluation object in advance and input these inputs into the model as input vectors to obtain the corresponding network output results. These data are the comprehensive evaluation results [15]. This network structure is suitable for multiple training and can get different output results. If a large error is found between the output value and the expected value, the threshold can be adjusted until the error can be accepted. After the training, the results obtained by the neural network can be analyzed qualitatively or evaluated quantitatively [16]. In the calculation process, BP neural network is generally through MATLAB software. It has strong calculation ability, is scientific and reliable, and reduces the error caused by human operation. BP neural network can carry out dynamic modeling, and this modeling is not fixed, does not need specific teaching methods or evaluation methods, and can deal with quantitative and qualitative indicators at the same time, so it has a wide range of applications.

BP neural network algorithm is divided into forward calculation and error calculation. Based on the power of three-layer network, it is assumed that there are *n* inputs, *q* outputs, and *p* hidden layer neurons. To initialize the network and learning parameters, in addition to the connection weight and neuron threshold, it is also necessary to set the learning rate and limit the error value. The training sample input vector and the expected output vector are covered. Calculate the input activation value and output value of the hidden layer. The calculation formula of input activation value of hidden layer is as follows:

(2) $$\begin{array}{c} s_{j}^{k}\;=\;\sum_{j=1}^{n}W_{ij}x_{i}^{k}-\vartheta_{j}. \end{array}$$

Calculate the input activation value of each neuron, and the formula is as follows:

(3) $$\begin{array}{c} l_{t}^{k}\;=\;\sum_{j=1}^{p}V_{jt}z_{j}^{k}-r_{t}, \end{array}$$

where *t*=1,2,3,…, *p*. In the BP learning algorithm, the global sum of squares error is used to judge the output result, and the calculation formula is as follows:

(4) $$\begin{array}{c} E_{k}=\sum_{t=1}^{q}\frac{{\left(y_{t}^{k}-c_{t}^{k}\right)}^{2}}{2}, \end{array}$$

where *E*_*k*_ is the sum of squares error. When the global sum of squares error is less than the predetermined value, the learning is considered to be over; otherwise, the convergence continues. If the maximum training coefficient is still not convergent, the network learning is still considered to be over. The expected value of neurons in the output layer is different from the actual output value, and their correction error can be expressed as

(5) $$\begin{array}{c} d_{t}^{k}=\left(y_{t}^{k}-c_{t}^{k}\right)\times c_{t}^{k}\times \left(1-c_{t}^{k}\right), \end{array}$$

where *y* represents the expected value, and *c* represents the actual output value. The correction error of each neuron in the hidden layer is calculated according to the calculated error, and the formula is as follows:

(6) $$\begin{array}{c} e_{j}^{k}=\left[\sum_{t=1}^{q}d_{t}^{k}v_{jt}\right]\times z_{j}^{k}\times \left(1-z_{j}^{k}\right). \end{array}$$

The adjustment formula is as follows:

(7) $$\begin{array}{c} V_{jt}\;\left(N+1\right)=V_{jt}\left(N\right)\;+\Delta V_{jt}\;\left(N+1\right),\\ W_{ij}\;\left(N+1\right)=W_{ij}\left(N\right)\;+\Delta W_{ij}\;\left(N+1\right), \end{array}$$

where *i* represents natural number, *N* represents learning times.

(8) $$\begin{array}{c} \Delta V_{jt}\left(N+1\right)=\eta d_{t}^{k}z_{j}^{k},\\ \Delta W_{ij}\left(N+1\right)=\eta e_{j}^{k}x_{j}^{k}. \end{array}$$

Adjust the output layer threshold and hidden layer neuron threshold. The adjustment formula is as follows:

(9) $$\begin{array}{c} r_{t}\;\left(N+1\right)\;=r_{t}\;\left(N\right)\;+\eta d_{t}^{k},\\ \theta_{j}\;\left(N+1\right)\;=\theta_{j}\;\left(N\right)\;+\eta e_{j}^{k}, \end{array}$$

where *η* is the learning rate. If the error end condition is not met, return to the previous step to continue training until the error meets the end condition.

BP neural network algorithm has its own defects. In order to ensure the convergence of the algorithm, the learning rate needs to set a fixed upper limit, so the convergence speed is slow. This algorithm adopts the gradient descent method, which gradually reaches the minimum error value along the vamp from a pilot area, but in fact, the space belongs to multidimensional surface, and local minimum points may appear [17]. At present, there is no clear theory on the selection of hidden layer nodes, which is generally based on empirical values and has great redundancy [18]. Therefore, we also need to optimize the algorithm and training set [19]. In the improved BP neural network, genetic algorithm needs to go through population initialization and integrate the input layer of BP network [20], integrating the hidden layer, connection weight, and output weight. The individuals are, respectively, corresponding to the four parts of BP neural network, differentiated and initialized to ensure the initial fitness [21]. The fitness function will affect the improvement effect. After the decoded individual obtains the predicted value, the test sample is used to predict the output value, and the root mean square error is used as the fitness function [22]. In the specific practice, we give priority to the selection roulette method. During the operation, because the individuals need to be encoded by real numbers, we should use the real number crossover method when performing the crossover operation. The specific crossover operations are as follows:

(10) $$\begin{array}{c} \left\{\begin{array}{c} a_{kj}=a_{kj}\left(1-b\right)+a_{lj}b,\\ a_{lj}=a_{lj}\left(1-b\right)+a_{kj}b, \end{array}\right. \end{array}$$

where *a* represents chromosome, and *b* represents random value, ranging from 0 to 1. Then, carry out mutation operation, and the formula is as follows:

(11) $$\begin{array}{c} a_{ij}=\left\{\begin{array}{cc} a_{ij}+\left(a_{ij}-a_{\max}\right)\times f\left(g\right), & r>0.5,\\ a_{ij}+\left(a_{\min}-a_{ij}\right)\times f\left(g\right), & r\leq 0.5, \end{array}\right. \end{array}$$

where *a*_max_, *a*_min_ represent the upper and lower limits of the gene, *r* is a random number, and *g* represents the number of iterations. When adjusting the weight of each layer of BP neural network, the error term is used to calculate and obtain the adjustment amount. When the operation result meets the end condition, the weight is automatically saved for evaluation and analysis [23]. If the calculation result does not meet the end condition, return the input vector again, and set the output vector until the end condition is met.

### 3.3. Training Samples and Evaluation Operation

The first part is the sample of entrepreneurship education, which is divided into two parts. According to each index value of innovation and entrepreneurship evaluation obtained above, calculate the index weight as the output sample value.

There are many factors affecting the evaluation results, and the evaluation indicators have an impact on the evaluation of ability. Combined with the set evaluation index model, we set the number of input layer nodes to 36. In addition, the hidden layer nodes need to be determined, which will affect the input and output results and test accuracy [24]. Use the following formula for determination:

(12) $$\begin{array}{c} q=\sqrt{n+m}+a. \end{array}$$

There are 12 nodes in the hidden layer of the model, and the number of output nodes is the evaluation result. In the evaluation, the number of nodes is 1, which is the comprehensive evaluation value. There is no relevant theoretical support at present; it needs to be trained. If the determination is too small, the samples cannot be identified, and too much will affect the learning time [25]. Therefore, based on ensuring the learning accuracy, if there is still no convergence within the specified training times, it will stop. Combined with the existing evaluation system, the hidden layer neuron is determined as 8.

**Figure 4:**
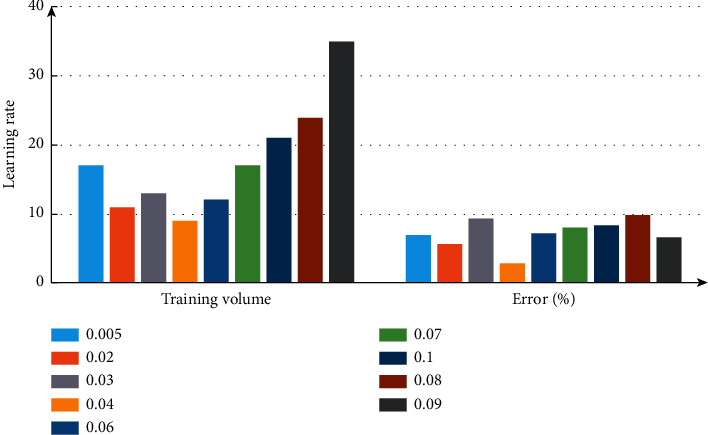
Comparative analysis of learning rate.

In the setting of learning rate, if it is too small, the training time will be prolonged, too large will affect the stability of the system, and it is not suitable to use the training samples with good initial performance. Therefore, the learning rate is set at 0.005∼0.09 The comparison results of learning rate are shown in Figure 4. Combined with the data in the figure, set the learning rate to 0.04. In order to avoid the situation that the training falls into the local minimum, the momentum factor value of 0.85 is introduced [26].

## 4. Result Analysis and Discussion

### 4.1. Algorithm Performance Analysis

**Figure 5:**
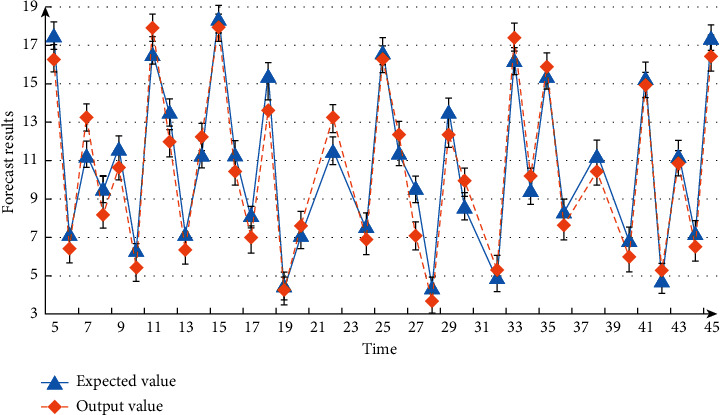
Measurement results of expected value and predicted value.

In this paper, the BP neural network optimized by genetic algorithm is programmed in MATLAB software. The genetic algorithm part makes the Sheffield genetic algorithm a box. The general function is used for fitting, and the performances of BP algorithm and improved algorithm based on genetic algorithm are compared and analyzed. Function selection *y*=*x*_1_^2^+*x*_2_^2^, randomly generate 2000 numbers, and compare and analyze the prediction results and time. When the genetic algorithm improves the BP neural network, the population size is set to 15, the evolution is 20 times, and the crossover probability is set to 0.3. The network parameter is set to 12, the output is 1, the maximum training times are 100, and the learning rate is set to 0.1. Compare and analyze the expected value and predicted value. The measurement results are shown in Figure 5. From the data in the figure, it can be seen that the output results of the algorithm proposed in this paper are basically similar to the expected results.

**Figure 6:**
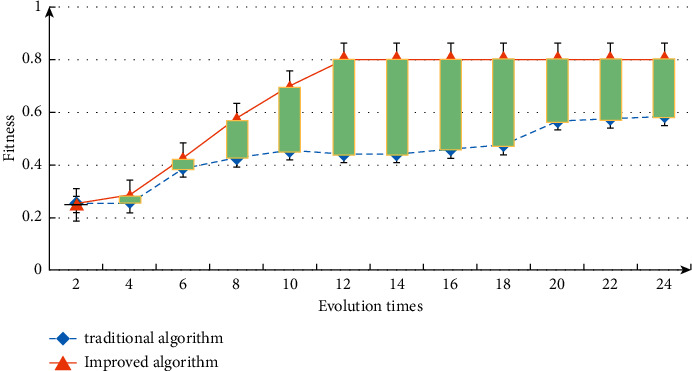
Individual fitness curve.

The analysis of the change curve of the best individual fitness is shown in Figure 6. From the data change in the figure, it can be seen that the traditional algorithm can not still determine whether it is stable after 20 iterations. The algorithm can obtain stable results after 14 iterations, indicating that the weight threshold of the improved algorithm is easier to obtain. The analysis of the change curve of the best individual fitness is shown in Figure 6. From the data change in the figure, it can be seen that the traditional algorithm can not still determine whether it is stable after 20 iterations. The algorithm can obtain stable results after 14 iterations, indicating that the weight threshold of the improved algorithm is easier to obtain.

**Figure 7:**
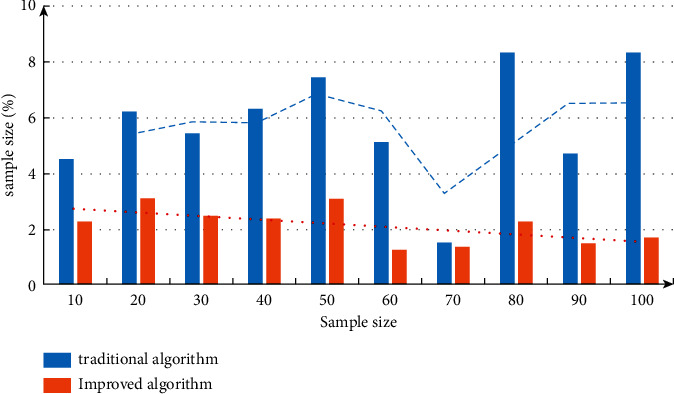
Comparative analysis of algorithm error.

Further compare and analyze the error changes. The measurement results are shown in Figure 7. Moreover, with the increase of sample size, the error change range of the algorithm is not very large, indicating that the performance of the algorithm is superior.

**Figure 8:**
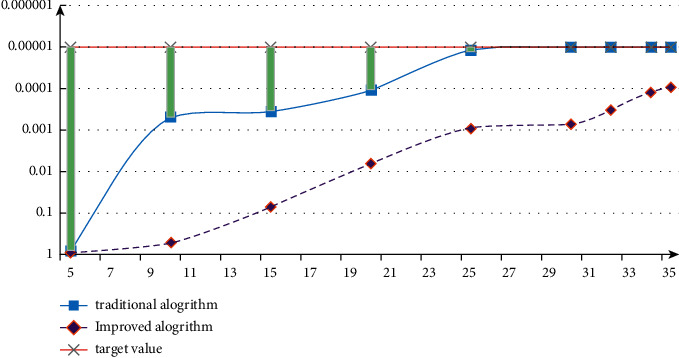
Variation curve between expected value and best quality.

Compare and analyze the change curves of the expected value and the best quality under the two algorithms. The measurement results are shown in Figure 8. From the data changes in the figure, it can be seen that using genetic algorithm to improve BP neural network can reduce the number of convergence, reduce the overall error, and improve the problem of slow convergence speed.

**Figure 9:**
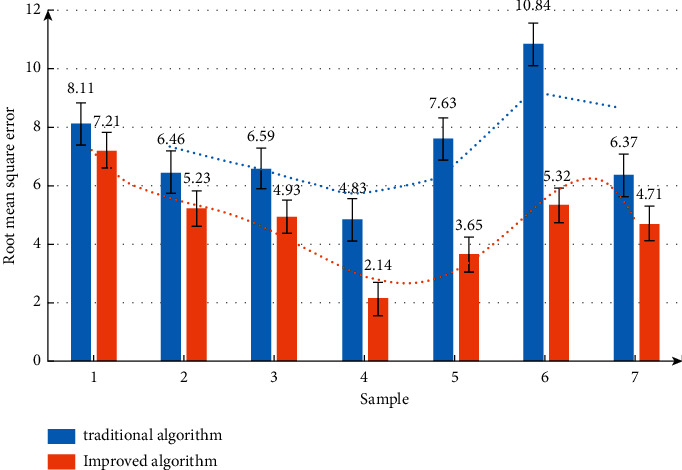
Comparison of test root mean square error.

Comparing the fitness value squared with the data before improvement, the analysis results are shown in Figure 9. It can be seen that the average mean square error has been further reduced after optimization by genetic algorithm, which shows that the optimization of BP neural network by genetic algorithm is successful.

### 4.2. Results

In the design and investigation of the questionnaire, due to different scores given by different people, it is necessary to remove the highest score and the lowest score and eliminate the data that are obviously inconsistent. The number of input layers is 8, and the number of input layers is 1. The data is normalized, and the input value is 0∼1. The maximum and minimum value method is used in the data processing. Using BP neural network model to analyze, the output values of the system are different. Because the evaluation is the teaching quality, the evaluation results are divided into five grades according to the specific data. The corresponding grade of neural network output 0.9∼1 is excellent, the corresponding grade of output value 0.8∼0.9 is good, the corresponding grade of output value 0.7∼0.8 is medium, the corresponding grade of output value 0.6∼0.7 is qualified, and the corresponding grade of output value less than 0.6 is unqualified.

In the modeling and simulation analysis, we used Matlab 7 0 software. In addition to calculation, the software also has visualization and programming functions. It can draw and has strong calculation ability. It can directly call functions and simulation tools and can easily study evaluation problems. According to the innovation and entrepreneurship evaluation system investigated and designed above, the weight and threshold of each index are preliminarily obtained. After normalization, the BP neural network model designed by the data determines the number of input layers, learning rate, and number of neurons carries out neural network training, stops convergence when the maximum training times or error value is reached, and inputs the test data for simulation analysis. Considering the use of the model to analyze the data, it is necessary to standardize the data and convert the scoring data in the percentage system into 0∼1 data for analysis. Statistically analyze the errors under different samples. The expected output values are 0.68, 0.75, and 0.72, respectively, the network output values are 0.680, 0.714, and 0.721, respectively, and the error values are 0.001, 0.004, and 0.002, respectively. It can be seen from the data that the data error is very small and can meet the needs.

According to the survey indicators and weights obtained from the questionnaire, the designed evaluation model is used for analysis. When the number of iterations reaches 76, stable results can be obtained, and the output value is 0.736, indicating that the evaluation of school is at the upper middle level, and there is still much room for improvement. Higher education school can encourage students to find employment in relevant fields, and students can gradually master relevant knowledge. In the teaching of relevant theories, practical teaching has also been paid attention to. Higher education school can start from practical links in combination with professional characteristics. The number of people participating in activities in higher education school is also increasing, and the quality of talents is improving.

## 5. Conclusion

At present, there are many researches on evaluation methods, among which BP neural network is widely used. The operation of BP neural network has no fixed requirements for data. In terms of calculation, it has strong adaptability, does not need specific mathematical expression, and can solve the problems of qualitative and quantitative evaluation and analysis. The evaluation in higher education school is not a simple linear problem analysis. Many nonlinear comprehensive evaluations can be solved by BP neural network to avoid the influence of human factors. Based on this, this paper studies the application of the improved neural network model in the evaluation of innovation and entrepreneurship ability of higher education school. This paper uses genetic algorithm to improve the BP learning algorithm and construct the BP neural network model. The simulation results show that after the genetic algorithm improves the BP neural network, the stable results can be obtained after 14 iterations. The weight threshold of the improved algorithm is easier to obtain, and the error prediction error is significantly less than that of the traditional algorithm. Moreover, with the increase of sample size, the error variation range of the algorithm is not very large, which can reduce the number of convergences, reduce the overall errors, and improve the problem of slow convergence speed. It should be pointed out that, in the analysis of different problems and in the evaluation system of innovation and entrepreneurship, the application of BP neural network will be affected by the sample size, threshold, and errors. In practical application, it is also necessary to reasonably select the improvement method.

However, with the gradual expansion of the application scope, BP neural network will also expose more and more shortcomings, for example, local minimization. BP neural network is an optimization method of local search. At the same time, the problem of slow convergence speed of BP neural network algorithm is not considered.

## Data Availability

The data used to support the findings of this study are available from the author upon request.
